# Efficacy of Permacol injection for perianal fistulas in a tertiary referral population: poor outcome in patients with complex fistulas

**DOI:** 10.1111/codi.15696

**Published:** 2021-05-16

**Authors:** Paul F. Vollebregt, Grietje J. Vander Mijnsbrugge, Charlotte B. H. Molenaar, Richelle J. F. Felt‐Bersma

**Affiliations:** ^1^ Department of Gastroenterology and Hepatology Amsterdam UMC Amsterdam Gastroenterology Endocrinology Metabolism Vrije Universiteit Amsterdam Amsterdam The Netherlands; ^2^ Proctos Kliniek Bilthoven The Netherlands

**Keywords:** anal fistula, collagen paste, perianal fistula, surgery

## Abstract

**Aim:**

Injection of Permacol collagen paste can be used as a sphincter‐sparing treatment for perianal fistulas. In a tertiary referral population we aimed to evaluate the efficacy of Permacol injection and the clinical and fistula‐related factors associated with recurrence.

**Method:**

This was a retrospective analysis of consecutive patients with perianal fistulas treated with Permacol injection at a specialist centre between June 2015 and April 2019. Endoanal ultrasonography was systematically reanalysed, blinded to treatment outcome. Rectovaginal, anovaginal and Crohn's disease fistulas were excluded. Healed fistulas were defined as absent anal symptoms and a closed external opening on physical examination at a minimum follow‐up of 6 months. Regression analyses were performed to identify factors associated with unhealed fistulas.

**Results:**

A total of 90 patients (51 men; median age 45 years) were analysed. Seventy‐two (80.0%) patients had complex perianal fistulas (greater than one‐third sphincter involvement or multiple tracts). After a single Permacol injection, fistulas were healed in 20 (22.2%) patients at 3 months follow‐up and in 18 (20.0%) patients at a median follow‐up of 30 months (interquartile range 17–37). Eight (11.1%) patients with unhealed fistulas had significant improvement in their symptoms. Complex fistulas were significantly associated with unhealed status [OR 3.53 (95% CI 1.12–11.09); *p* = 0.031]. Twenty patients underwent subsequent Permacol injections, which were successful in six (30.0%) patients after one (*n* = 3) or two (*n* = 3) additional injections.

**Conclusion:**

This largest study to date in patients with mainly complex perianal fistulas, demonstrated that the efficacy of a single Permacol injection was only 20%. Complex fistulas were associated with a poor outcome.

**What does this paper add to the literature?**

In this largest study to date to report on Permacol injection for mainly complex perianal fistulas, we found a healing rate of 20.0% at a median follow‐up of 30 months. In contrast to previous studies in less complex fistulas, these results suggest that Permacol injection is not effective in complex fistulas.

## INTRODUCTION

Perianal fistulas are defined as abnormal tracts between the anorectum and the perineum. A variety of concepts describing their underlying pathophysiology have been described [1, 2, 3, 4]. The incidence of fistula‐in‐ano in European countries is estimated to be between 1.2 and 2.8 per 10 000 per annum [5].

Symptoms from perianal fistulas may have a profound impact on a patient's quality of life [6], and the majority of patients require surgery to eradicate the primary tract. In low fistulas, fistulotomy is the most commonly performed procedure and is considered the gold standard by most surgeons [7], resulting in low recurrence rates [8, 9]. Nevertheless, such interventions may jeopardize anal sphincter function in patients with high or complex fistulas, as a larger part of sphincter muscle is involved [10, 11]. Indeed, several sphincter‐sparing techniques have been developed as alternative treatment options [12, 13, 14, 15, 16]. Biomaterials have also been shown to have a limited impact on anal function, although recurrence rates remain significant and do not improve upon traditional surgical techniques [17, 18].

Permacol is a recently introduced biomaterial in the treatment algorithm for perianal fistulas, consisting of an acellular cross‐linked porcine dermal collagen matrix suspension. It is injected into the fistula tract, enabling the fistula to heal without damaging the anal sphincter complex. The MASERATI100 study showed a healing rate of 53.5% in a large population of patients with primary or recurrent fistulas at 12 months follow‐up [19]. To date, only a small number of other studies have been published on the efficacy of Permacol, all suggesting similar healing rates [20, 21, 22, 23, 24]. Nonetheless, outcome data in complex anal fistulas are scarce, and the available evidence is based on studies which are limited in sample size (i.e. fewer than 50 patients) [21, 22, 24]. Hence, we analysed a series of consecutive patients referred to a specialist centre for treatment of mainly complex perianal fistulas with the aim of evaluating the efficacy of Permacol injection and the clinical and fistula‐related factors associated with unhealed fistula status after Permacol injection.

## METHOD

### Study sample

A retrospective review of medical records was performed for consecutive patients (aged ≥18 years) who underwent perianal fistula treatment with Permacol injection at a tertiary referral centre (Proctos Clinic, Bilthoven, The Netherlands) between June 2015 and April 2019. Prior to surgery, all patients attended a clinic at which a structural history was undertaken by the operating surgeon, evaluating medical conditions and previous (anorectal) surgical events. Subsequently, the operating surgeon performed a clinical examination and endoanal ultrasonography with the patient in left lateral position. Patients with recto‐ or anovaginal fistulas or Crohn's disease were excluded to reduce heterogeneity of the study population. Follow‐up of at least 6 months after Permacol injection was required for study inclusion.

### Three‐dimensional endoanal ultrasonography and fistula characterization

Three‐dimensional images of the anal canal were acquired by endoanal ultrasonography with a 10–16 MHz transducer (Hawktype 2050, B‐K Medical, Naerum, Denmark). Three per cent hydrogen peroxide was introduced into the external opening of the fistula using an intravenous cannula in order to visualize the fistula tract. To reduce reporting bias, all endoanal ultrasonography recordings were systematically reanalysed by a single senior investigator with over 30 years' experience (RFB) who was blinded to preoperative assessment and treatment outcome. Fistulas were classified according to the Parks classification [2]. Transsphincteric fistulas were subclassified as being low (lower third of the sphincter complex), mid (middle third of the sphincter complex) or high (upper third of the sphincter complex). Other systematically collected fistula characteristics were the number of tracts, height (in mm) of the internal opening measured from the anal verge and the distance (in mm) of the furthest external opening measured from the anodermal junction. All fistulas were further classified as being simple (low, and single tract) or complex (mid or high, or multiple tracts). Finally, patients were classified as presenting with a primary or recurrent fistula, with the latter characterized as those with a previously failed surgical intervention.

### Surgical procedure

Prior to the procedure a Microlax enema was used, and a combination of 1 g cefazoline and 500 mg metronidazole was administered intravenously. The patient was placed in the lithotomy position and the internal fistula opening was identified using a Czerny forceps. If a seton was present it was removed. The fistula tract was debrided with a curette, followed by rinsing of the fistula tract with 0.9% NaCl. If required, the internal opening was debrided from hypertrophic tissue. The internal opening was sutured using Vicryl 2.0 full‐thickness sutures without tying the sutures. A flexible cannula sheath was introduced in the external opening and the Permacol™ collagen paste (Medtronic) was installed in the fistula tract until the paste was visualized at the internal opening. The sutures were tied when the tract was completely filled, i.e. when the paste was visible at both the internal and external openings. Debridement of the external opening was performed and all hypertrophic tissue was excised. The external opening was approximated loosely with one suture, so that the paste stayed inside the fistula tract. A sterile dressing was applied.

### Outcome measures

The efficacy of a single Permacol injection was evaluated in all patients at (a) the standard clinic appointment 3 months after surgery (i.e. short‐term efficacy) and (b) throughout the follow‐up period until the final physical follow‐up appointment. A healed fistula was defined clinically as the absence of anal symptoms (e.g. discharge, discomfort, pain) and a closed external opening confirmed on physical examination by the operating surgeon. Unhealed fistulas were further classified as those with (a) worsened/similar anal symptoms, (b) improved anal symptoms (both based on direct questioning at the clinic appointment) or (c) further surgical interventions performed to achieve fistula healing.

### Data analysis

Patient demographics and fistula classification were described using proportions or median (interquartile range, IQR). Fistula healing after a single Permacol injection was presented as the number and proportion of patients with a healed fistula at 3 months and at the end of follow‐up. Fistulas in patients treated with further Permacol injections were classified as unhealed, and subsequent proportions of fistula healing were described separately. Univariate binary regression analyses [odds ratio (OR), 95% confidence interval (95% CI)] were performed to identify factors associated with unhealed fistulas. A value of *p* < 0.05 was considered statistically significant. Data were analysed using SPSS version 21.0 software (SPSS Inc., Chicago, IL, USA).

## RESULTS

### Study sample and fistula characterization

A total of 104 patients underwent Permacol injection for anal fistulas during the study period. Of these, five (4.8%) patients with anovaginal fistulas and five (4.8%) patients with Crohn's disease were excluded. A further four patients were excluded due to a follow‐up period of less than 6 months, leaving a study sample of 90 patients [51 men (56.7%)]; the median age was 45 years (IQR 36–53). Patient characteristics, previously performed fistula related interventions and fistula classification are shown in Table 1. A total of 80 (88.9%) patients had previously undergone fistula‐related surgery with a median of three procedures (IQR one to four), or 302 procedures in total. Seton drainage was most frequently performed [in 66 (73.3%) of patients]. Fistulas were classified as recurrent in 38 (42.2%) patients. Transsphincteric high fistulas were most common (*n* = 43; 47.8%) and 18 (20.0%) consisted of multiple tracts. Overall, this resulted in 72 (80.0%) fistulas being classified as complex.

**TABLE 1:** Patient characteristics, previously performed fistula‐related surgery and fistula characterization in the study sample (90 patients)

	No. of patients (%)
Sex	
Female	39 (43.3)
Male	51 (56.7)
Age (years), median (IQR)	45 (36–53)
Smoker^a^	
No	73 (82.0)
Yes	16 (18.0)
Previous fistula‐related surgery	80 (88.9)
Seton drainage	66 (73.3)
Incision and drainage	59 (65.6)
Fistulotomy	15 (16.7)
Mucosa advancement flap	14 (15.6)
Ligation of intersphincteric fistula tract	12 (13.3)
Fistula laser closure	6 (6.7)
Plug	3 (3.3)
Miscellaneous	10 (11.1)
Recurrent fistula	
Yes	38 (42.2)
No	52 (57.8)
Fistula classification	
Type	
Intersphincteric	16 (17.8)
Transsphincteric low	13 (14.4)
Transsphincteric mid	18 (20.0)
Transsphincteric high	43 (47.8)
Height of internal opening from anal verge (mm)	
1–9	18 (20.0)
10–19	48 (53.3)
20–29	17 (18.9)
30–39	5 (5.6)
40–50	2 (0.2)
Distance furthest external opening from anodermal junction (mm)^b^	
0–20	23 (27.1)
21–40	50 (58.8)
41–60	11 (12.9)
61–80	1 (1.2)
Multiple tracts	18 (20.0)
Complex	72 (80.0)

^a^ Unknown in one patient.

^b^ Unknown in five patients.

### Perioperative data

Prior to Permacol injection, seton drainage was performed in 56 (62.2) patients for a median period of 6 months (IQR 4–8 months). The Permacol injections were performed by six surgeons, with a median of 14 procedures per surgeon. The median time for the procedure was 22 min (IQR 19–30 min). Two patients developed postoperative abscesses. One patient underwent seton drainage of the abscess 8 days post‐Permacol injection. The abscess drained spontaneously in the other patient. No other perioperative adverse events were recorded.

### **Short**‐**term efficacy: 3 months follow‐up**

At 3 months follow‐up, the perianal fistula was healed in 20 (22.2%) patients with a closed external opening and without any anal symptoms. Of the 70 patients with anal symptoms at 3 months follow‐up, 61 (87.1%) reported no improvement in symptoms and 9 (12.9%) reported temporary improvement only. Physical examination demonstrated an external fistula opening in 60 (85.7%) patients with an unhealed fistula. In the 10 patients with a closed fistula opening, reported anal symptoms were discharge (*n* = 9) and pain (*n* = 1).

### Efficacy

Perianal fistulas were healed in 18 (20.0%) patients after a single Permacol injection at a median follow‐up of 30 months (IQR 17–37 months). Of the 72 patients with an unhealed fistula, 59 (81.9%) underwent further surgery, with the next procedure being performed at a median of 7 months after initial Permacol injection (IQR 5–17) (Table S1 in the Supporting Information). Of the remaining patients with unhealed fistulas, 8 (11.1%) had significant symptom improvement and hence had not undergone further surgical procedures. No further surgery was performed in 5 (6.9%) patients despite significant complaints.

Demographics, the main fistula characteristics and associations with unhealed fistula status after a single Permacol injection at a median follow‐up of 30 months are shown in Table 2. Simple fistulas were unhealed in 11/18 (61.1%) and complex fistulas in 61/72 (84.7%) patients; complex fistulas were significantly associated with unhealed status [OR 3.53 (95% CI 1.12–11.09); *p* = 0.031]. The rate of persisting fistulas was high after previously performed mucosal advancement flap (*n* = 13/14; 92.9%), ligation of intersphincteric fistula tract procedure (*n* = 10/12; 83.3%) and fistula laser closure (*n* = 6/6; 100%), although these factors were not statistically associated with outcome. Inter‐surgeon healing rate differences were not statistically significant (*p* = 0.124).

**TABLE 2:** Association of demographics and main fistula characteristics with unhealed fistulas after a single Permacol injection using univariate binary regression analysis

Variable	Unhealed fistula (%)	Odds ratio (95% CI)	*p*‐value
Sex			
Female	33/39 (84.6)	Reference	
Male	39/51 (76.5)	0.59 (0.20–1.75)	0.342
Age (years)	‐	0.97 (0.93–1.02)	0.204
Smoker^a^			
No	60/73 (82.2)	Reference	
Yes	12/16 (75.0)	0.65 (0.18–2.34)	0.510
Recurrent fistula			
No	39/52 (75.0)	Reference	
Yes, after:	33/38 (86.8)	2.20 (0.71–6.82)	0.172
Mucosal advancement flap	13/14 (92.9)	3.75 (0.46–30.72)	0.219
Ligation of intersphincteric fistula tract	10/12 (83.3)	1.29 (0.26–6.49)	0.758
Fistulotomy	13/15 (86.7)	1.76 (0.36–8.63)	0.484
Fistula type			
Intersphincteric	11/16 (68.9)	Reference	
Transsphincteric low	9/13 (61.5)	1.02 (0.21–4.98)	0.978
Transsphincteric mid	16/18 (72.2)	3.64 (0.60–22.2)	0.162
Transsphincteric high	36/43 (83.7)	2.34 (0.62–8.85)	0.211
Multiple tracts			
No	57/72 (79.2)	Reference	
Yes	15/18 (83.3)	1.32 (0.34–5.15)	0.693
Complex			
No	11/18 (61.1)	Reference	
Yes	61/72 (84.7)	**3.53 (1.12–11.09)**	**0.031**
Seton drainage pre‐Permacol			
No	30/34 (88.2)	Reference	
Yes	42/56 (75.0)	0.40 (0.12–1.34)	0.136

Bold indicates significant values.

^a^ Unknown in one patient.

**FIGURE 1:**
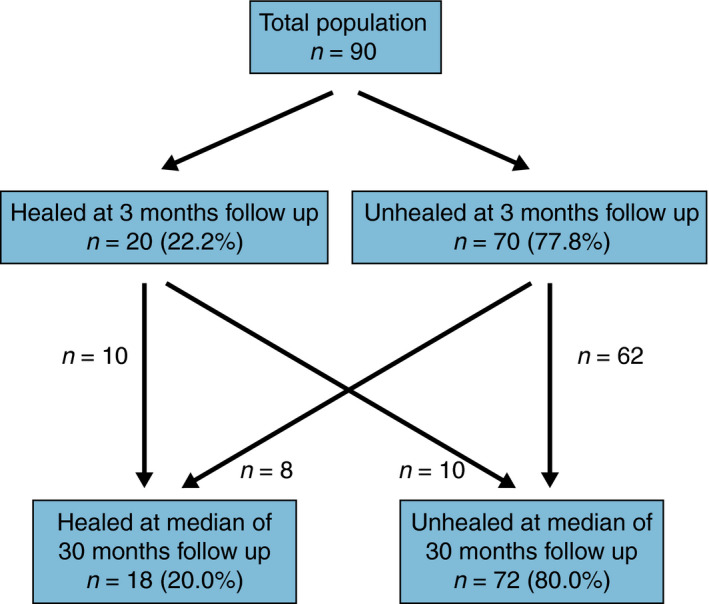
Proportions of patients with a healed fistula at a median of 30 months follow‐up, and their fistula status at 3 months follow‐up

Of all patients with a healed fistula at a median of 30 months follow‐up, only 10/18 fistulas (55.6%) were already healed at 3 months follow‐up (Figure 1). In the other eight patients, the fistula healed between 3 and 6 months in four patients, between 6 and 9 months in two patients; the timing of fistula healing was unknown in the other two patients. Conversely, 10/72 (13.9%) patients with an unhealed fistula at a median of 30 months follow‐up had a closed external opening without the presence of anal symptoms at 3 months after surgery.

A total of 20 patients with an unhealed fistula after a single Permacol injection underwent subsequent Permacol injections. This was successful in six (30.0%) patients: after one (three patients) or two (three patients) additional Permacol injections, evaluated at a median follow‐up of 20 months (IQR 11–30) after the final Permacol injection.

## DISCUSSION

In 90 consecutive patients undergoing Permacol injection for perianal fistulas, we found a healing rate of 22.2% (*n* = 20) at 3 months follow‐up and 20.0% (*n* = 18) at a median follow‐up of 30 months. Complex fistulas were associated with a success rate of only 15.3%, while fistula healing was achieved in 38.9% of patients with a simple fistula. This is the largest study to date reporting on Permacol injection for mainly complex fistulas, with the longest follow‐up period.

The outcome of Permacol injection in our cohort was poor compared with other studies (summary of previous studies with a minimum of ≥10 patients; Table 3) [19, 20, 21, 22, 23, 24]. The proportion of patients with healed fistulas was almost three‐fold lower than in the MASERATI100 study at 12 months follow‐up (56.7%) [19]. Their definition of fistula healing was identical to that in our study (absence of anal symptoms and a closed external opening confirmed on physical examination), making it possible to compare like with like. However, the proportion of patients with a fistula with over one‐third sphincter involvement in our study was almost two‐fold higher than in the MASERATI100 study (66.3% vs. 35.6%). Indeed, a shorter fistula tract, which usually corresponds with less sphincter involvement, was associated with favourable outcome in the MASERATI100 study. Additionally, 18 (20.0%) patients in our study had multiple tracts, which might explain the poor outcome of our study as they are generally associated with recurrence [25], although such fistulas were excluded in the MASERATI100 study [19]. Nonetheless, our population reflects daily clinical practice in a tertiary care setting, which usually consists of a higher proportion of patients with complex fistulas.

**TABLE 3:** Previous studies on Permacol injection for perianal fistulas with10 or more patients

Author	Year	Country	Study type	*n* (%)	Female (%)	Age (years)	Recurrent (%)	High fistula (>1/3) (%)	Multiple tracts (%)	Crohn's disease (%)	Rectovaginal (%)	Healing rate (%)	Follow‐up (months)
Hammond et al. [23]	2011	UK	Prospective	13	4 (30.8)	43 (35–53)^a^	2 (15.4)	7 (53.8)	0	0	0	7 (53.8)	29 (4–43)^b^
Fabiani et al. [22]	2017	Italy	Prospective	21	8 (38.1)	48 (22–72)^b^	3 (14.3)	18 (85.7)	7 (33.3)	1 (4.8)	0	10 (47.6)	12
Giordano et al. [19]	2017	European	Prospective	100	30 (30.0)	48 (20–78)^b^	39 (39.0)	26/73 (35.6)	0	0	0	53/99 (53.5)	12
Bayrak et al. [20]	2018	Turkey	Retrospective	31	17 (54.8)	45 (25–68)^b^	6 (19.4)	Unknown	4 (12.9)	0	0	24 (77.4)	13 (11–15)^b^
Brunner et al. [21]	2019	Germany	Prospective	30	19 (63.0)	46 (17–78)^a^	30 (100)	Unknown	0	12 (40.0)	6 (20.0)	17 (57.0)	≥6 in 30 patients ≥12 in 24 patients
Schiano di Visconte et al. [24]	2019	Italy	Retrospective	46	21	41 (24–78)^b^	20	46 (100)	0	0	0	23 (50.0)	24 (1–25)^b^
Current study	2021	The Netherlands	Retrospective	90	39 (43.3)	45 (20–75)^b^	38 (42.2)	61 (67.8)	18 (20.0)	0	0	18 (20.0)	30 (9–60)^b^

^a^ Mean (range).

^b^ Median (range).

Other studies reported efficacy rates much like the MASERATI100 study, with a range between 47.6% and 57.0% (Table 3) [21, 22, 23, 24]. Only one study reported a much higher healing rate of 77.4% [20]. Nevertheless, patients in our cohort were more likely to have recurrent fistulas, a higher internal opening or multiple tracts and the follow‐up period was longer than in the other studies, which might explain the difference in proportions of fistula healing. Alternative strategies to improve success rates, such as a combined procedure of Permacol injection and an advancement flap, have been studied by creating a ‘barrier’ at the internal fistula opening to avoid contamination of the paste. In a consecutive series of 24 patients, at a mean follow‐up of 14 months, healing rates of 82.3% (14/17) in cryptoglandular and 57.1% (4/7) in inflammatory bowel disease‐related fistulas were reached [26]. These initial results require confirmation from further studies.

We acknowledge the following study limitations. Firstly, the retrospective design has its known disadvantages. Although important study variables (systematic and blinded fistula characterization using endoanal ultrasonography) and endpoints (standard 3 months after operation and at the end of follow‐up clinic appointments) were consistently evaluated, the study was not protocol based. Hence, the surgical technique and reporting of clinical outcomes may be subject to variability between surgeons. A second limitation concerns sampling. Data were derived from a nonrandomized intervention at a single tertiary referral centre, which limits generalizability to other centres and particularly to populations with less complex fistulas. Thirdly, the primary endpoint was based on clinical evaluation of fistula healing, and further evaluation using endoanal ultrasonography or MRI was not performed at follow‐up in patients without complaints. Although unlikely given the long follow‐up period, this might overestimate the proportion of patients with a closed fistula. The primary endpoint in most other Permacol studies was also based on clinical assessment [19, 20, 21, 22, 24]. Fourthly, a larger population is needed to adjust for confounders (e.g. smoking) in the evaluation of factors associated with recurrence (we restricted the regression analyses to univariate rather than multivariate models). Fifth, observer bias should be mentioned as one of the limitations as outcome was evaluated by different surgeons (each patient was evaluated by his or her own operating surgeon). Lastly, quality of life measures evaluating patients' experiences were missing, and outcome was solely based on direct questioning about complaints and physical examination. Recently, consensus groups have sought to develop core outcome sets for the evaluation of cryptoglandular [27] and Crohn's disease fistulas [28] in order to involve all stakeholders (researchers, patients and other healthcare professionals) in the measurement of treatment outcome. Indeed, once validated, these measurements will be likely to improve the evaluation of fistula surgery in future studies.

Many studies evaluating surgical techniques in perianal fistulas suggest high initial success rates, although this tends to decrease when more data are published. This can possibly be explained by the fact that recently implemented techniques are mainly trialled in simple cases and only subsequently used in more complex disease. Our data show a poor outcome compared with previous studies, implying that Permacol injection, which is not an inexpensive treatment option (approximately €550 per injection), is not suitable in more complex perianal fistulas. Success rate after one or two additional Permacol injections increased to 30%; however, the number of subjects undergoing subsequent injections was small. In patients with simple fistulas with a priori risk for faecal incontinence, Permacol may be an alternative sphincter‐sparing treatment option.

## CONCLUSION

This study in patients with mainly complex perianal fistulas demonstrated that the efficacy of a single Permacol injection was only 20%. Complex fistulas (more one third of sphincter complex involvement or multiple tracts) were associated with a poor outcome. Permacol injection should not be considered a treatment option in complex fistulas.

## CONFLICT OF INTEREST

PFV, GJVM, CBHM and RJFF‐B have no conflicts of interest.

## AUTHOR CONTRIBUTIONS

All authors conceived the study design. PFV collected the study data, analysed the data and wrote the manuscript. All authors edited the manuscript and approved the final version.

## AUTHOR CONTRIBUTIONS

All authors conceived the study design. Paul F vollebregt collected the study data, analysed the data and wrote the manuscript. All authors edited the manuscript and approved the final version.

## ETHICAL APPROVAL

The study was approved by the Medical Ethical Committee of the VU University Medical Centre (reference number 2013/21).

## Supporting information

Table S1. Further surgery in 59 patients with a recurrent fistula after single Permacol injection.

## Data Availability

The data that support the findings of this study are available from the corresponding author upon reasonable request.
