# Supplementary material for: Efficacy of Permacol injection for perianal fistulas in a tertiary referral population: poor outcome in patients with complex fistulas
Source: Colorectal Dis. 2021 May 16;23(8):2119–26. doi: 10.1111/codi.15696 (PMC8453864; doi:10.1111/codi.15696)
Supplement: Supplementary file 1 — Table S1. Further surgery in 59 patients with a recurrent fistula after single Permacol injection. [file CODI-23-2119-s001.docx]

**Supplementary Table 1.** Further surgery in 59 patients with a recurrent fistula after single Permacol injection.

| **Variable** | **N (%)** |
| --- | --- |
| Description of further procedure  Permacol  Seton drainage  Fistulotomy  Abscess drainage  Fistula laser closure  Mucosa advancement flap  Ligation of intersphincteric fistula track (LIFT)  Debridement fistula  Stem cells | 31  22  11  8  7  6  2  2  1 |
| Number of further procedures  1  2  3  4 | 33 (55.9)  21 (36.0)  4 (6.8)  1 (1.7) |
| Fistula status after further surgery  Healed  Median follow-up (months, IQR)  Persistent  Persistent, anal symptoms improved  Recently performed last procedure | 25 (42.4)  7 (4 – 10)  19 (32.2)  3 (5.1)  12 (20.3) |
